# Serum calcium and magnesium levels in women presenting with pre-eclampsia and pregnancy-induced hypertension: a case–control study in the Cape Coast metropolis, Ghana

**DOI:** 10.1186/s12884-014-0390-2

**Published:** 2014-11-20

**Authors:** Richard Kobina Dadzie Ephraim, Derick Nii Mensah Osakunor, Seth Wiredu Denkyira, Henrietta Eshun, Samuel Amoah, Enoch Odame Anto

**Affiliations:** Department of Medical Laboratory Technology, University of Cape Coast, Cape Coast, Ghana; Department of Molecular Medicine, School of Medical Sciences, College of Health Sciences, Kwame Nkrumah University of Science and Technology, Kumasi, Ghana; Laboratory Department, University Health Services, University of Cape Coast, Cape Coast, Ghana

**Keywords:** Calcium, Electrolytes, Magnesium, Pregnancy-induced hypertension, Pre-eclampsia

## Abstract

**Background:**

Hypertensive disorders of pregnancy are important causes of morbidity and mortality. The levels of calcium (Ca^2+^) and magnesium (Mg^2+^) in pregnancy may implicate their possible role in pregnancy-induced hypertension. This study assessed serum Ca^2+^ and Mg^2+^ levels in women with PIH (pregnancy-induced hypertension) and PE (pre-eclampsia), compared to that in normal pregnancy.

**Methods:**

This case–control study was conducted on 380 pregnant women (≥20 weeks gestation) receiving antenatal care at three hospitals in the Cape Coast metropolis, Ghana. This comprised 120 women with PIH, 100 women with PE and 160 healthy, age-matched pregnant women (controls). Demographic, anthropometric, clinical and obstetric data were gathered using an interview-based questionnaire. Venous blood samples were drawn for the estimation of calcium and magnesium.

**Results:**

Systolic blood pressure (SBP) and diastolic blood pressure (DBP) were significantly raised in women with PIH (*p* < 0.0001) and PE (*p* < 0.0001). Women with hypertensive disorders (PE and PIH) had significantly lower serum calcium and magnesium levels than those in the control group (*p* < 0.0001 each). Of those with PIH, SBP correlated positively with BMI (r = 0.575, *p* < 0.01) and Ca^2+^ correlated positively with Mg^2+^ (r = 0.494, *p* < 0.01). This was similar amongst the PE group for SBP and BMI as well as for Ca^2+^and Mg^2+^ but was not significant. Multivariate analysis showed that women aged ≥40 years were at a significant risk of developing PIH (OR = 2.14, *p* = 0.000).

**Conclusion:**

In this study population, serum calcium and magnesium levels are lower in PIH and PE than in normal pregnancy. Mineral supplementation during the antenatal period may influence significantly, the occurrence of hypertensive disorders in pregnancy.

## Background

Hypertensive disorders of pregnancy, such as pre-eclampsia (PE) and pregnancy-induced hypertension (PIH) are a major cause of maternal morbidity [1]. The incidence rate of pre-eclampsia stands at 3–10% globally, [2] with a 7% rate reported amongst Ghanaian pregnant women [3,4]. Taking into account numerous studies conducted, the aetiology of this condition remains unknown [5], although factors such as obesity, diabetes, calcium (Ca^2+^) deficiency [6], advanced maternal age, oxidative stress, placental ischemia, genetics and immune maladaptation have been implicated [7].

PIH develops due to pregnancy and regresses after delivery. It is a known cause of premature delivery, intrauterine growth restriction (IUGR), placental abruption, foetal death, and numerous adverse pregnancy outcomes. Previous history of pre-eclampsia, pre-existing diabetes, multiple pregnancies, nulliparity and advanced maternal age (≥40 years) have been reported to increase the risk of PIH and thus PE [7-9].

Previous reports have considered the association between hypertensive disorders of pregnancy (PE and PIH) and serum electrolytes, particularly Ca^2+^ and magnesium (Mg^2+^) [10-13]. It has been reported that there are reduced levels of Ca^2+^ and Mg^2+^ in pre-eclampsia [12,13]. Meanwhile, a recent report from Nigeria has indicated that apart from raised levels of sodium in pre-eclampsia, Ca^2+^ and Mg^2+^ were within normal reference intervals [14]. To date, a study on Ca^2+^ and Mg^2+^ levels amongst women presenting with PE and PIH is non-existent in Ghana, although the use of magnesium sulphate remains the treatment of choice, especially in eclampsia. Are there changes in levels of these electrolytes and are these changes a contributing factor to the causation of hypertensive disorders of pregnancy in Ghana? It is therefore expedient to address this gap, with the aim of providing preliminary data that could influence the prevention and management of PE and PIH in Ghana.

We assessed the levels of serum Ca^2+^ and Mg^2+^ in women presenting with PE and PIH, compared to that in normal pregnancy. We further assessed factors that may contribute to an increased risk of PIH and PE in Ghana.

## Methods

### Study design/site

This multicenter case–control study with consecutive sampling was conducted in the Cape Coast metropolis, Ghana, from December 2013 to May, 2014. The hospitals involved in the study included, the Cape Coast Teaching Hospital (CCTH), University of Cape Coast Hospital (UCCH) and the Cape Coast metropolitan hospital. Cape Coast covers a total land mass of 9,826 square kilometres (2223.9843 acres) with an estimated population of 1,805,488 [15].

### Ethical consent

The study was approved by the Institutional Review Board of the University of Cape Coast (IRB-UCC) and authorities of the selected hospitals. Participation was voluntary and written informed consent was obtained from each participant.

### Eligibility criteria

Pregnant women with gestational age 20 weeks or more were eligible participants for this study. Participants in good health, normotensive and without dipstick (reagent strip) proteinuria were enrolled as controls. Participants with elevated blood pressure (≥140/90 mmHg) with or without dipstick proteinuria (≥“+”) were enrolled as cases (PE and PIH respectively). Pregnant women with chronic hypertension, on antihypertensive therapy, eclampsia, diabetes, autoimmune disease and renal disease were excluded from the study.

### Study population

Three hundred and eighty (380) participants (≥20 weeks gestation) were involved in the study. This included 160 age-matched, pregnant normotensive women, 120 pregnant women with PIH and 100 pregnant women with PE, receiving antenatal care at the selected centres. Participants with high blood pressure (≥140/90 mmHg) on two occasions, at least four hours apart, with visible dipstick proteinuria (≥“+”), were categorised as PE and those without proteinuria were categorised as PIH [1,16]. A standard interview-based questionnaire was used to obtain data on demography, clinical and family history.

### Anthropometric measurements

Height (to the nearest 0.1 cm) without shoes was measured with a wall-mounted ruler. Weight (to the nearest 0.1 kg) in light clothing was measured with a bathroom scale (Zhongshan Camry Electronic Co. Ltd, Guangdong, China). BMI was calculated using the formula; weight (kg)/height (m^2^).

### Blood pressure measurement

Trained personnel measured the blood pressure of participants in accordance with recommendations of the American Heart Association [17]. Repeated measurements were taken within 5–10 minutes rest interval and the mean value was recorded as the blood pressure.

### Urinalysis

Participants provided 10–20 ml of freshly voided early morning urine in clean, wide-mouthed, leak-proof containers. Urinalysis was performed using a dipstick, semi-qualitative method as per manufacturer’s instructions (CYBOW™ DFI Co Ltd, Gimhae-City, Republic of Korea). Proteinuria in participants with pre-eclampsia was defined as the presence of urinary protein in concentrations ≥ “+”, using the semi-quantitative colour scale on the urine reagent dipstick [16].

### Blood sample collection and biochemical analysis

After observing an overnight fast, 4 ml of venous blood sample was taken from each participant into serum separator tubes (Becton Dickinson, Rutherford, NJ).

Blood samples were allowed to clot and centrifuged at 3000 rpm for 3–5 minutes within 30 minutes of sample collection. Serum Ca^2+^ and Mg^2+^ was estimated immediately, using an automated electrolyte analyser (FT-1000 Automatic Chemical analyser, Fortune company Limited, Chengdu, China).

### Statistical analysis

Data was entered and stored in Microsoft Excel and analysed using SPSS version 16.0 (SPSS Inc. Chicago). Categorical variables were analysed using Chi-square and Fischer’s tests whilst continuous variables were analysed using the unpaired t-test. Multiple groups were compared using One-way ANOVA coupled with Dunnett multiple comparisons. Pearson’s correlation and multiple logistic regression analyses were performed to determine the relationship between variables and to identify independent factors associated with hypertensive disorders (PIH and PE) respectively. In all statistical tests, a value of p <0.05 was considered significant.

## Results

As shown in Table 1, there was no significant difference in maternal and gestational ages between the three groups compared. Mean blood pressure (SBP and DBP) was significantly higher in women with PIH (SBP = 155, DBP = 101; *p* < 0.0001) and PE (SBP = 152 DBP = 102; *p* < 0.0001), compared to those in the control group (SBP = 112, DBP = 65).

**Table 1:** **Demographic, clinical and obstetric related characteristics of the study population**

**Variables**	**Control**	**α** ***p*** **-value**	**PIH**	**β** ***p*** **-value**	**PE**	**γ** ***p*** **-value**
	**(n = 160)**		**(n = 120)**		**(n = 100)**	
Age *(years)*	30.70 ± 4.24	0.0809	30.03 ± 6.74	0.2817	32.28 ± 8.58	0.0560
SBP *(mmHg)*	112.50 ± 8.40	**< 0.0001**	155.17 ± 10.21	0.2935	152.60 ± 7.09	**< 0.0001**
DBP *(mmHg)*	65.25 ± 6.40	**< 0.0001**	101.63 ± 7.84	0.5133	102.24 ± 6.81	**< 0.0001**
BMI *(kgm* ^*−2*^ *)*	28.25 ± 5.67	0.4827	29.70 ± 11.43	0.9381	29.04 ± 7.61	0.4484
Gestational age *(weeks)*	31.92 ± 4.69	0.6320	31.40 ± 4.27	0.4993	30.72 ± 2.84	0.2518
***Gravidity n (%)***						
Primigravida	80 (50.0)	0.9626	56 (46.7)	0.8949	56 (56.0)	0.7885
Secundigravida	36 (22.5)	0.2865	48 (40.0)	0.0675	12 (12.0)	0.5693
Multigravida	44 (27.5)	0.1133	16 (13.3)	0.2484	32 (32.0)	0.9364
***Parity n (%)***						
Nulliparous	88 (55.0)	0.9904	68 (56.7)	0.9693	60 (60.0)	0.9246
Primiparous	40 (25.0)	0.9451	36 (30.0)	0.4769	16 (16.0)	0.6286
Multiparous	32 (20.0)	0.4346	16 (13.3)	0.5936	24 (24.0)	0.9748
***Contraceptive use n (%)***						
Yes	68 (42.5)	0.3922	32 (26.7)	0.9103	32 (32.0)	0.6989
***History of Abortion n (%)***						
Yes	40 (25.0)	0.2793	12 (10.0)	0.9723	12 (12.0)	0.4437
***New paternity n (%)***						
Yes	40 (25.0)	0.7022	20 (16.7)	0.2610	36 (36.0)	0.6377

Values are in mean ± SD and frequency (proportion) where appropriate. Comparison between proportions was done using Fischer’s exact test. One-way ANOVA coupled with Dunnett multiple comparisons was used to compare multiple groups. α P-value (comparison between control & PIH); βp-value (comparison between PIH & PE); γp-value (comparison between controls & PE). PIH: Pregnancy-induced hypertension; PE: Pre-eclampsia, SBP: Systolic blood pressure; DBP: Diastolic blood pressure. Data in boldface indicate statistical significance.

**Figure 1:**
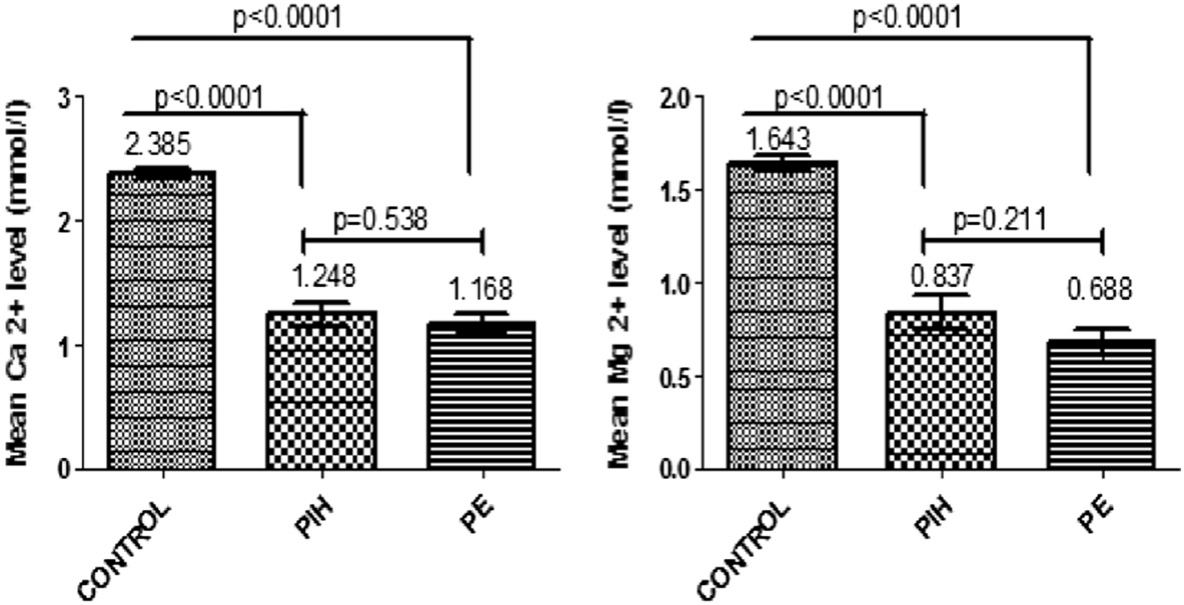
**Mean Ca** ^**2+**^ **and Mg** ^**2+**^ **levels in PIH, PE and the control group.** PIH: Pregnancy-induced Hypertension, PE: Pre-eclampsia, Ca^2+^: Calcium, Mg^2+^: Magnesium. P < 0.05 was considered significant for statistical comparisons using the t-test.

In Figure 1, there was a significantly lower mean serum Ca^2+^ and Mg^2+^ levels amongst pregnant women with hypertensive disorders (PE and PIH) than in the corresponding control group (*p* < 0.0001). There was no significant difference in the mean serum Ca^2+^ (*p* = 0.538) and Mg^2+^ (*p* = 0.211) levels between women with PIH and PE although lower in women with PE.

All women with PIH and PE had hypocalcaemia (100% each). The proportion of women with PIH and PE with hypomagnesaemia was 86.7% and 100% respectively. Of the 160 pregnant women in the control group, 8 (5%) had hypocalcaemia and 24 (15%) had hypomagnesaemia. One hundred and fifty-two (152) pregnant women, representing 40%, of the total population were overweight. Of this, 64 (40%) were from the control group, 36 (30%) were women with PIH and 52 (52%) were women with PE [Table 2].

**Table 2:** **Prevalence of overweight, obesity, hypocalcaemia and hypomagnesaemia amongst study participants**

**Variables**	**Controls (n = 160)**	**PIH (n = 120)**	**PE (n = 100)**	**Total (Prevalence)**
**BMI** ***(kg/m*** ^***2***^ ***)***				
BMI >25	64 (40.0%)	36 (30.0%)	52 (52.0%)	152 (40.0%)
BMI <25	96 (60.0%)	84 (70.0%)	48 (48.0%)	228 (60.0%)
Obese (≥30)	52 (32.5%)	44 (36.7%)	24 (24.0%)	120 (31.6%)
Non-obese (<30)	108 (67.5%)	76 (63.3%)	76 (76.0%)	260 (68.4%)
**Ca** ^**2+**^ ***(mmol/l)***				
Low (<2.1)	8 (5.0%)	120 (100.0%)	100 (100.0%)	228 (60.0%)
Normal (2.1-2.8)	152 (95.0%)	0 (0.0%)	0 (0.0%)	152 (40.0%)
**Mg** ^**2+**^ ***(mmol/l)***				
Low (<1.5)	24 (15.0%)	104 (86.7%)	100 (100.0%)	228 (60.0%)
Normal (1.5-2.0)	136 (85.0%)	16 (13.3%)	0 (0.0%)	152 (40.0%)

BMI = Body Mass Index, Ca^2+^ = Calcium, Mg^2+^ = Magnesium, PIH: Pregnancy-induced hypertension; PE: Pre-eclampsia.

There was a significant positive correlation between the DBP and SBP (r = 0.370, *p* < 0.05), BMI and SBP (r = 0.434, *p* < 0.01) and between Ca^2+^and Mg^2+^ (r = 0.841, *p* < 0.01) amongst the control group (Tables 3 and 4). Of those with PIH, SBP correlated positively with BMI (r = 0.575, *p* < 0.01), with a similar observation between serum Ca^2+^and Mg^2+^ levels (r = 0.494, *p* < 0.01) (Table 3). A similar trend of positive association between SBP and BMI as well as between Ca^2+^and Mg^2+^ was observed amongst women with PE but this was not significant [Table 4].

**Table 3:** **Pearson’s correlation co-efficient of association between variables for the control (lower left-hand side) and PIH (upper right-hand side) groups**

**Variable**	**Age**	**SBP**	**DBP**	**GA**	**BMI**	**Ca** ^**2+**^	**Mg** ^**2+**^
**Age**		0.047	0.100	−0.215	0.020	−0.181	0.059
**SBP**	0.194		0.115	0.149	**0.575** ^**^	−0.314	−0.083
**DBP**	0.201	**0.370** ^*^		−0.321	0.129	−0.221	0.158
**GA**	−0.085	−0.015	−0.166		0.329	0.038	−0.234
**BMI**	0.303	**0.434** ^**^	0.068	0.011		0.045	0.151
**Ca** ^**2+**^	0.179	−0.181	−0.130	0.023	0.020		**0.494** ^**^
**Mg** ^**2+**^	0.147	−0.201	−0.111	−0.043	−0.113	**0.841** ^**^	

*Correlation is significant at the 0.05 level (2-tailed). **Correlation is significant at the 0.01 level (2-tailed). GA: Gestational Age; BMI: Body Mass Index: DBP: Diastolic Blood Pressure; SBP: Systolic Blood Pressure. Data in boldface indicate statistical significance.

**Table 4:** **Pearson’s correlation co-efficient of association between variables for the control (lower left-hand side) and PE (upper right-hand side) groups**

**Variable**	**Age**	**SBP**	**DBP**	**GA**	**BMI**	**Ca** ^**2+**^	**Mg** ^**2+**^
**Age**		0.197	0.267	0.346	−0.239	0.054	−0.118
**SBP**	0.194		0.254	−0.066	0.095	0.011	0.284
**DBP**	0.201	**0.370** ^*^		0.012	−0.209	0.078	0.236
**GA**	−0.085	−0.015	−0.166		0.309	−0.139	−0.190
**BMI**	0.303	**0.434** ^**^	0.068	0.011		−0.387	−0.356
**Ca** ^**2+**^	0.179	−0.181	−0.130	0.023	0.020		0.106
**Mg** ^**2+**^	0.147	−0.201	−0.111	−0.043	−0.113	**0.841** ^**^	

*Correlation is significant at the 0.05 level (2-tailed). **Correlation is significant at the 0.01 level (2-tailed). GA: Gestational Age; BMI: Body Mass Index: DBP: Diastolic Blood Pressure; SBP: Systolic Blood Pressure. Data in boldface indicate statistical significance.

The effect of different variables on the risk of developing PIH and PE is shown in Table 5. The only significant factor in this study that increased pregnant women’s risk of PIH, as determined by multivariate analysis was age. Pregnant women aged 40 years and above were about two times more likely to develop PIH than women under 25 years of age (OR = 2.14, *p* = 0.000).

**Table 5:** **Multivariate logistic regression model for risk factors associated with PIH and PE**

**Variable**	**PIH**		**PE**	
	**OR (95% CI)**	***p*** **-value**	**OR (95% CI)**	***p*** **-value**
***Age group***				
< 25*	1		1	
25-29	0.82 (0.22-2.98)	0.757	0.53 (0.13-2.27)	0.395
30-34	0.44 (0.09-2.09)	0.305	0.27 (0.04-1.73)	0.165
35-39	4.00 (0.59-27.25)	0.157	4.8 (0.68-33.80)	0.115
≥ 40	2.14 (0.35-13.27)	**0.000**	5.14 (0.47-14.87)	0.278
***Gravidity***				
Primigravida*	1		1	
Secundigravida	1.91 (0.63-5.73)	0.252	0.48 (0.11-2.08)	0.324
Multigravida	0.52 (0.14-1.97)	0.335	1.04 (0.33-3.24)	0.948
***Parity***				
Nulliparous*	1		1	
Primiparous	1.17 (0.39-3.50)	0.786	0.59 (0.16-2.22)	0.433
Multiparous	0.65 (0.17-2.51)	0.529	1.10 (0.32-3.82)	0.881
***BMI***				
Normal*	1		1	
Overweight	0.62 (0.19-2.02)	0.427	1.49 (0.43-5.12)	0.527
Obese	0.93 (0.29-3.01)	0.905	0.85 (0.21-3.39)	0.813
***Contraceptive use***				
No*	1		1	
Yes	0.49 (0.18-1.37)	0.174	0.64 (0.22-1.82)	0.399
***Abortion***				
No*	1		1	
Yes	0.33 (0.08-1.34)	0.122	0.41 (0.10-1.66)	0.212
***New paternity***				
No*	1		1	
Yes	0.60 (0.18-1.99)	0.403	1.69 (0.57-5.00)	0.345
***Malaria***				
No*	1		1	
Yes	2.92 (0.50-17.15)	0.235	0.00	-

BMI = Body Mass Index, PIH: Pregnancy-induced hypertension; PE: Pre-eclampsia. *Reference. Data in boldface indicate statistical significance.

## Discussion

Hypertensive disorders of pregnancy are associated with increased morbidity and mortality, especially during delivery [1]. Our study was conducted to assess the levels of serum Ca^2+^ and Mg^2+^ in pregnant women with PE and PIH compared to that in normal pregnancy. It also identified factors that may contribute to an increased risk of PIH and PE. To date, no such study has been conducted in Ghana. Our results showed that levels of serum Ca^2+^ and Mg^2+^ was significantly reduced in women with PE and PIH and advanced maternal age (≥40) was associated with a higher risk of developing PIH. The present study adds to implications on the aetiology of hypertension in pregnancy in Ghana and may influence prevention and treatment through mineral supplementation during the antenatal period.

Hypertensive disorders are the commonest medical complications that develop during pregnancy, characterized by an increase in blood pressure. In accordance with presentation of the condition, the present study showed that systolic and diastolic blood pressure was significantly raised in women with PIH and PE [9,18].

Serum calcium and magnesium are very important for metabolism at the cellular level and are vital for muscle contraction, cell death and neuronal activity [13], making it very essential in pregnancy. The observation of low Ca^2+^ and Mg^2+^ levels is in agreement with other studies on hypertensive disorders in pregnancy [10,12,13,19]. A probable theory to this observation may be that when serum calcium levels decreased, the levels of intracellular calcium increased, leading to constriction of smooth muscles in blood vessels and therefore increased vascular resistance [20-22], culminating in a raised systolic and diastolic blood pressure. Furthermore, previous reports suggest that altered calcium homoeostasis, as exhibited by increased calcium excretion, is associated with higher blood pressure levels [23]. Low serum calcium levels may also increase blood pressure by stimulating parathyroid hormone and rennin release, which in turn increases intracellular calcium in smooth muscle, leading to vasoconstriction [24]. The observation is further supported by the 2011 WHO recommendation, which found a higher risk of pre-eclampsia in pregnant women with low dietary intake of calcium and recommended supplementation for such women [25]. This implies that calcium levels may play a role in hypertensive disorders in pregnancy.

Golmohammad Lou *et al*. [26] have disputed the role of calcium and trace elements in high blood pressure, for that matter, in pre-eclampsia. They explained that, although slightly lower, there was no significant difference in calcium and magnesium levels between women with pre-eclampsia and normal, healthy pregnant women [26]. This however, is slightly contentious as magnesium supplementation during treatment of pre-eclampsia and seizures, has been shown to oppose calcium-dependent arterial constriction and may antagonize the increase in intracellular calcium concentration. A Cochrane review as well as recommendations by the WHO on prevention and management of pre-eclampsia and eclampsia have consistently supported that supplementation of these minerals in pregnancy is associated with a significant reduction in the risk of pre-eclampsia [25,27,28]. Therefore, exemptions to the fact that low calcium and magnesium levels exist in such conditions may not be justified.

The observed low levels of magnesium in women with PIH and PE could be due to decreased dietary intake, increased clearance by the kidneys, haemodilution due to expansion of the extracellular space and increased consumption of minerals by the growing foetus [13]. This together with lowered calcium levels play a role in the development of hypertensive disorders in pregnancy. Other researchers have proposed that a reduction in the level of extracellular Mg^2+^ causes partial membrane depolarization and decreased repolarisation along with opening of Ca^2+^ membrane channels, leading to an intracellular Ca^2+^ shift. Furthermore, the existing increase in the foetal Ca^2+^ demand may also block bone resorption of Ca^2+^ with a concurrent intracellular pull [19,29]. This phenomenon produces vasoconstriction together with an increase in the blood pressure, as seen in PIH and PE.

The effect of Mg^2+^ and Ca^2+^ in the pathogenesis of hypertensive disorders in pregnancy is supported by the observed positive correlation between magnesium and calcium in all participating groups, although not significant amongst those with PE.

Further correlation analysis showed a positive association between BMI and SBP in all groups and was significant amongst both controls and those with PIH but not PE. This relationship is similar to findings from other studies [30-33]. The observed association is thought to be as a result of the role of obesity-mediated inflammation in the pathogenesis of PIH [34].

Multivariate logistic regression showed that pregnancy in advanced maternal age (≥40 years) was associated with about a two-fold and five-fold increase in the odds of developing PIH and PE respectively. This was however significant for PIH but not PE. This observation is similar to that observed in other studies [8,9,35] but contrary to reports of existing significant risk of PE in a much younger age group (≤21 years) [36]. Acknowledging that PIH and PE may only be different manifestations of the same disease, there is some evidence that these conditions may be distinct in pathophysiology [37]. Furthermore, the increased risk of hypertensive disorders in pregnancy with increasing age has been suggested to be associated with the biological changes that occur with ageing [9]. These biological changes may interact differently with the pathophysiological processes of PIH and PE, hence yielding different outcomes. In a systematic review of controlled trials [8], women aged ≥40 had about two times the risk of developing pre-eclampsia, whether they were primiparous or multiparous [38]. This review however found that most studies failed to address differences between participants at baseline; hence, only one study was taken into account. Much therefore remains to be determined concerning age and hypertensive disorders in pregnancy, especially PE. That notwithstanding, caution must be exercised in assessing risk of PE in advanced maternal age.

How much decline in levels of serum Mg^2+^ and Ca^2+^ is associated with an increased risk of hypertension in pregnancy? At what stage do these changes begin to manifest as a rise in blood pressure and proteinuria? Can these electrolytes exhibit a predictive ability for PIH and PE? To clarify these, it is important to conduct further studies.

## Conclusion

In this study population, levels of serum calcium and magnesium are significantly low in women with PIH and PE than in normal pregnancy. Increased maternal age was associated with an increased risk of PIH but not PE. This may suggest a cause and effect relationship between these electrolytes and hypertension in pregnancy. It helps to understand the pathophysiological process of hypertension in pregnancy, and to establish and enhance existing preventive strategies for the condition. Mineral supplementation during the antenatal period may influence significantly, the occurrence of hypertensive disorders in pregnancy.

### Limitations

Due to limited resources, we were unable to use more sensitive and reliable quantitative methods of detecting proteinuria. Dietary patterns was not determined, thus the impact of inadequate intake of these minerals as well as the possible confounding effect of diet remains. There is limitation on the generalization of the study findings due to consecutive sampling technique used. Findings from the study however remain relevant and add to evidence on the subject matter.

## Abbreviations

PIH: Pregnancy-induced Hypertension
PE: Pre-eclampsia
Mg^2+^: Magnesium
Ca^2+^: Calcium
SBP: Systolic blood pressure
DBP: Diastolic blood pressure
BMI: Body mass index

## References

[1] Magee LA, Pels A, Helewa M, Rey E, von Dadelszen P, Hypertension Guideline C (2014). Diagnosis, evaluation, and management of the hypertensive disorders of pregnancy: executive summary. J Obstet Gynaecol Can.

[2] Osungbade KO, Ige OK (2011). Public health perspectives of preeclampsia in developing countries: implication for health system strengthening. J Pregnancy.

[3] Obed S, Aniteye P (2007). Pregnancy following eclampsia: a longitudinal study at korle-bu teaching hospital. Ghana Med J.

[4] Turpin CA, Ahenkorah L, Owiredu WKBA, Laing EF, Amidu N (2008). The Prevalence of the Metabolic Syndrome Among Ghanaian Pregnancy-Induced Hypertensive Patients Using the World Health Organisation and the National Cholesterol Education Program III Criteria. J Med Sci.

[5] Onder C, Seyma H, Yusuf T, Mehmet H, Remzi G (2003). Cerebrospinal fluix nitric oxide level changes in preeclampsia. Eur J Obstet Gynaecol Reprod Biol.

[6] Sibai BM (1996). Treatment of hypertension in pregnant women. N Engl J Med.

[7] Serhal PF, Craft I (1987). Immune basis for pre-eclampsia evidence from oocyte recipients. Lancet.

[8] Duckitt K, Harrington D (2005). Risk factors for preeclampsia at antenatal booking: systematic review of controlled studies. BMJ.

[9] Owiredu WKBA, Ahenkorah L, Turpin C, Amidu N, Laing EF (2012). Putative risk factors of pregnancy induced hypertension among Ghanaian pregnant women. J Med Biomed Sci.

[10] Abdellah A, Abdrabo AA (2014). Assessment of serum calcium, magnesium, copper and zinc levels in Sudanese pregnant women with preeclampsia. Glo Adv Res J Med Sci.

[11] Kumru S, Aydin S, Simsek M, Sahin K, Yaman M, Ay G (2003). Comparison of serum copper, zinc, calcium, and magnesium levels in preeclamptic and healthy pregnant women. Biol Trace Elem Res.

[12] Sayyed KA, Sonttake AS (2013). Electrolytes status in preeclampsa. OIIRJ.

[13] Sandip S, Asha K, Paulin G, Hiren S, Gagandeip S, Amit V (2013). A comparative study of serum uric acid, calcium and magnesium in preeclampsia and normal pregnancy. JAdvanc Res Biol Sci.

[14] Adewolu OF (2014). Serum sodium, potassium, calcium and magnesium in women with pregnancy induced hypertension and preeclampsia in Oredo local Government, Benin Metropolis: A pilot study. Afr J Med Health Sci.

[15] GSS: **2012 Population and Housing Census.** In *ᅟ.* Accra, Ghana: Ghana Statistical Service; 2012:1–11.

[16] American College of O, Gynecologists, Task Force on Hypertension in P (2013). Hypertension in pregnancy. Report of the American College of Obstetricians and Gynecologists' Task Force on Hypertension in Pregnancy. Obstet Gynecol.

[17] Kirkendall WM, Burton AC, Epstein FH, Freis ED (1967). Recommendations for human blood pressure determination by sphygmomanometers. Circulation.

[18] Idogun ES, Imarengiaye CO, Momoh SM (2007). Extracellular calcium and magnesium in preeclampsia and eclampsia. Afr J Reprod Health.

[19] Indumati V, Kodliwadmath M, Sheela M (2011). The role of serum electrolytes in pregnancy induced hypertension. J Clin Diagnostic Res.

[20] Ingec M, Nazik H, Kadanali S (2006). Urinary calcium excretion in severe preeclampsia and eclampsia. Clin Chem Lab Med.

[21] Lopez-Jaramillo P (2000). Calcium, nitric oxide, and preeclampsia. Semin Perinatol.

[22] Szmidt-Adjide V, Vendittelli F, David S, Bredent-Bangou J, Janky E (2006). Calciuria and preeclampsia: a case–control study. Eur J Obstet Gynecol Reprod Biol.

[23] Kesteloot H, Tzoulaki I, Brown IJ, Chan Q, Wijeyesekera A, Ueshima H, Zhao L, Dyer AR, Unwin RJ, Stamler J, Elliott P (2011). Relation of urinary calcium and magnesium excretion to blood pressure: The International Study Of Macro- And Micro-nutrients And Blood Pressure and The International Cooperative Study On Salt, Other Factors, And Blood Pressure. Am J Epidemiol.

[24] Selina A, Shelina B, Sultana F (2011). Calcium and Zinc deficiency in preeclamptic women. J Bangladesh Soc Physiol.

[25] ᅟ**Recommendations for Prevention and Treatment of Pre-eclampsia and Eclampsia.** In *ᅟ.* ; ᅟ [http://www.who.int/mediacentre/factsheets/fs311/en/] 23741776

[26] Golmohammad Lou S, Amirabi A, Yazdian M, Pashapour N (2008). Evaluation of Serum Calcium, Magnesium, Copper, and Zinc Levels in Women with Pre-eclampsia. Iran J Med Sci.

[27] Hofmeyr GJ, Lawrie TA, Atallah AN, Duley L (2010). Calcium supplementation during pregnancy for preventing hypertensive disorders and related problems. Cochrane Database Syst Rev.

[28] Hofmeyr GJ, Lawrie TA, Atallah AN, Duley L, Torloni MR (2014). Calcium supplementation during pregnancy for preventing hypertensive disorders and related problems. Cochrane Database Syst Rev.

[29] Farzin L, Sajadi F (2012). Comparison of serum trace element levels in patients with or without pre-eclampsia. J Res Med Sci.

[30] Agyemang C, Oudeman E, Zijlmans W, Wendte J, Stronks K (2009). Blood pressure and body mass index in an ethnically diverse sample of adolescents in Paramaribo, Suriname. BMC Cardiovasc Disord.

[31] Droyvold WB, Midthjell K, Nilsen TI, Holmen J (2005). Change in body mass index and its impact on blood pressure: a prospective population study. Int J Obes (Lond).

[32] Tesfaye F, Byass P, Wall S (2009). Population based prevalence of high blood pressure among adults in Addis Ababa: uncovering a silent epidemic. BMC Cardiovasc Disord.

[33] Tesfaye F, Nawi NG, Van Minh H, Byass P, Berhane Y, Bonita R, Wall S (2006). Association between body mass index and blood pressure across three populations in Africa and Asia. J Hum Hypertens.

[34] Getahun D, Ananth CV, Oyelese Y, Chavez MR, Kirby RS, Smulian JC (2007). Primary preeclampsia in the second pregnancy: effects of changes in prepregnancy body mass index between pregnancies. Obstet Gynecol.

[35] Stone P, Cook D, Hutton J, Purdie G, Murray H, Harcourt L (1995). Measurements of blood pressure, oedema and proteinuria in a pregnant population of New Zealand. Aust N Z J Obstet Gynaecol.

[36] Anorlu RI, Iwuala NC, Odum CU (2005). Risk factors for pre-eclampsia in Lagos, Nigeria. Aust N Z J Obstet Gynaecol.

[37] Fisher KA, Luger A, Spargo BH, Lindheimer MD (1981). Hypertension in pregnancy: clinical-pathological correlations and remote prognosis. Medicine.

[38] Bianco A, Stone J, Lynch L, Lapinski R, Berkowitz G, Berkowitz RL (1996). Pregnancy outcome at age 40 and older. Obstet Gynecol.

